# Analysis of a city‐wide COVID‐19 prevention strategy for aged‐care facilities during third and fifth waves of COVID‐19 in Kyoto City, Kyoto, Japan

**DOI:** 10.1111/irv.12981

**Published:** 2022-03-09

**Authors:** Miki Nagao, Yasufumi Matsumura, Masaki Yamamoto, Koh Shinohara, Satomi Yukawa, Taro Noguchi, Yasuhiro Tsuchido, Takeshi Ikeda

**Affiliations:** ^1^ Department of Clinical Laboratory Medicine Kyoto University Graduate School of Medicine Kyoto Japan; ^2^ Department of Clinical Laboratory, Department of Infection Prevention Kyoto University Hospital Kyoto Japan; ^3^ Public Health and Welfare Bureau of Kyoto City Kyoto Japan

**Keywords:** COVID‐19, elderly, infection prevention, mass testing, vaccination

## Abstract

**Background:**

During the third wave of the COVID‐19 pandemic at the end of 2020, clusters occurred frequently in aged‐care facilities (ACFs), which put pressure on the medical field in Japan. Based on this experience, Kyoto University and Kyoto City collaborated to promote a citywide COVID‐19 prevention strategy to prevent the spread of COVID‐19 within ACFs. The aim of this study was to clarify the effect of the prevention strategy among ACFs in Kyoto City during the third and fifth waves of the pandemic.

**Methods:**

During the study period, the following measures were adopted as the prevention strategy in all ACFs: (1) active polymerase chain reaction (PCR) mass testing and facility‐wide testing when a single case was identified, (2) implementation of strategies to prevent transmission within a facility, and (3) vaccination program for ACFs.

**Results:**

Of the 1,144 facilities subjected to the mass testing, 71.0% participated in the whole program including active PCR testing. The remainder participated in the rest of the programs. The prevalence of ACF‐related COVID‐19 cases among total COVID‐19 cases in Kyoto City decreased from 7.9% in the third wave to 4.1% in the fourth wave and 2.1% in the fifth wave. The incidence of clusters and proportion of severe elderly cases also decreased during the study period.

**Conclusions:**

A city‐wide multidisciplinary effort including PCR mass testing and a vaccination program in cooperation with a university and local administrative office successfully reduced the clusters and transmission in ACFs in Kyoto City, Japan.

## 1 INTRODUCTION

COVID‐19 has had a disproportionate impact on vulnerable populations such as the elderly, particularly residents of aged‐care facilities (ACFs).^1^, ^2^, ^3^, ^4^, ^5^, ^6^ To date, a large amount of evidence has been accumulated on effective strategies to contain and prevent the spread of COVID‐19 in ACFs.^7^, ^8^, ^9^, ^10^ Assessing the real‐world impact of infection prevention measures in nursing homes is complicated but, at the same time, critical if the aim is to resume welfare systems and health care delivery systems as they were prior to the pandemic.

In the middle of the COVID‐19 pandemic, ACFs accounted for a large proportion of total deaths attributed to the virus in Japan as well.^1^, ^11^ In Japan, the Ministry of Health, Labour and Welfare has been taking the lead in issuing guidance for ACFs. However, during the third wave of the pandemic, COVID‐19 outbreaks occurred frequently in medical institutions and ACFs, and there were also high incidences of morbidity and mortality among the elderly, which put pressure on the medical field in Japan. Based on this experience, the local government of Kyoto City (public health) and Kyoto University Hospital (KUH; a university hospital with infectious disease and clinical laboratory specialists) collaborated to promote a city‐wide COVID‐19 prevention strategy to prevent the spread of COVID‐19 within ACFs. The aim of this study was to clarify the effect of the strategy among ACFs during the third and fifth waves of the pandemic in Kyoto City, Kyoto Japan.

## 2 METHODS

### 2.1 The subjects of the study

Kyoto is a city of 1.45 million people, with 28.4% of the population aged 65 or over. It is an aging society where the aging rate has doubled in the past 5 years. The subjects of this study were users and staff of ACFs including both residential and day‐care facilities for the elderly in Kyoto City, Kyoto, Japan.

### 2.2 Study period

The study period was from the third (2020/12/1–2021/2/28) to fifth (2021/6/25–2021/9/30) waves. Because there is no official definition of the third wave, the beginning of the third wave was defined as the day when 30 new cases were identified, and the end of the wave was tentatively defined as February 28, when the number of new infections per day was consistently below 15. The definitions of fourth and fifth waves were based on those of Kyoto Prefecture, from March 1 to June 24, and June 25 to September 30, respectively.

### 2.3 Study design

#### 2.3.1 Prospective, observation study

### 2.4 Multifaceted infection‐prevention measures

This project was originally started as quarantine measure to prevent the introduction of COVID‐19 to ACF. During a preliminary intervention, an educational project for ACF staff launched and a vaccination program for the elderly began. Following the preliminary intervention with a small number of facilities during the third wave, the target facilities were expanded to include all ACFs (both residential facilities and day‐care centers) in the city, and the following measures were adopted from the beginning of April (fourth wave): (1) mass testing; (2) implementation of code of conduct and infection prevention techniques involving staff, elderly, and their families to prevent infection both in the community and within the facilities; and (3) vaccination for the elderly and staff of ACFs, which was of the highest priority along with health care professionals in Japan.

(1) Mass testing for residents and staff and facility‐wide testing strategies when a single case was identified

Saliva samples for universal serial polymerase chain reaction (PCR) testing were submitted to KUH (in March and April) and a testing company (from April to September) weekly from the end of the third to end of the fifth waves. When a screening PCR test was positive (Step 1), a confirmation test was performed at KUH (Step 2) simultaneously with facility‐wide testing. The reasons for adopting the two‐step policy were as follows: A false‐positive result is even more of an issue when the test is performed for asymptomatic individuals and the pre‐test probability is low, as it was in this case. In addition, reports of notification must be submitted by a physician in Japan, but the testing company undertaking mass testing did not have such a policy. Eventually, the mass testing ended when the facility finished vaccinating the residents and staff.

(2) Implementation of strategies to prevent transmission within a facility

As a measure to prevent the introduction of the disease into the facility, educational materials were distributed to facility staff, new users, and their families regarding the request to self‐isolate for 2 weeks after using the facility, sick‐leave policy, and daily health monitoring. In addition, we provided materials on the appropriate use of personal protective equipment and conducted a training session on infection control using Zoom as an enhanced education program. During the third wave, we realized that there had not been enough guidance to meet the level of infection control at ACFs. As such, in order to fit the actual ACF settings, we modified the manuals and education materials that were used in KUH and distributed them to each facility.

(3) Vaccination program

Pfizer‐BioNTech COVID‐19 vaccines were distributed to elderly and staff of ACFs on a priority basis starting in the middle of April from residential‐type facilities to day‐care centers and including nursing helpers.

As such, the infection prevention strategy was expanded from the quarantine measure using a PCR mass testing only to the “shielding measures” by educational programs and vaccination program to prevent the spread of COVID‐19 within in the facilities.

### 2.5 Evaluation of the effect of multifaceted intervention

To evaluate the effect of the infection prevention strategy, we analyzed the number of clusters per month, number of COVID‐19 cases related to ACFs, and proportion of severe cases between the third wave and fourth and fifth waves. A cluster was defined as an outbreak within a facility involving more than five cases. Major lineages of SARS‐CoV‐2 in Kyoto City during the study period are also shown in the Table 1.

**TABLE 1:** SARS‐COV‐2 testing and COVID‐19 cases in ACFs in Kyoto City, from the third to fifth waves in 2020–2021

	The third wave	The fourth wave	The fifth wave
	Without mass testing	Serial mass testing	
	2020/12/1–2021/02/28	2021/03/1–2021/06/24	2021/06/25–2021/09/30
Major lineage of SARS‐CoV‐2	B.1.1.214	Alpha(B.1.1.7‐like)	Delta(B.1.617‐2‐like)
Total number of ACF‐related COVID‐19 cases (sporadic + cluster)	363	147	210
Proportion of cluster‐related COVID‐19 cases	75.2%	60.5%	42.4%
Average number of cluster episodes in a month	7.3	2.6	3.7
Average number of COVID‐19 cases in each cluster	12.4	9.9	8.1
Average number of PCR tests in each cluster case	71	141.1	157.3
Number of facilities subject to universal active screening		1,706	1,144
Number of facilities conducting universal active screening (proportion, %)		1,177 (67.2%)	786 (71.0%)
Total number of universal active screening tests		182,684	211,693
Number of screening‐positive cases		39	33
Positivity rate for screening test (%)		0.020%	0.016%
Number of confirmed COVID‐19 cases		21	20
False‐positive rate (%)		66.7%	70.0%

Abbreviations: ACF, aged‐care facility; PCR, polymerase chain reaction.

### 2.6 Ethics approval

The Ethics Committee of Kyoto University Graduate School and Faculty of Medicine approved this study (R2379) and waived the need to obtain informed consent from each patient.

## 3 RESULTS

### 3.1 The results of mass tests and trend of COVID‐19 cases

**FIGURE 1:**
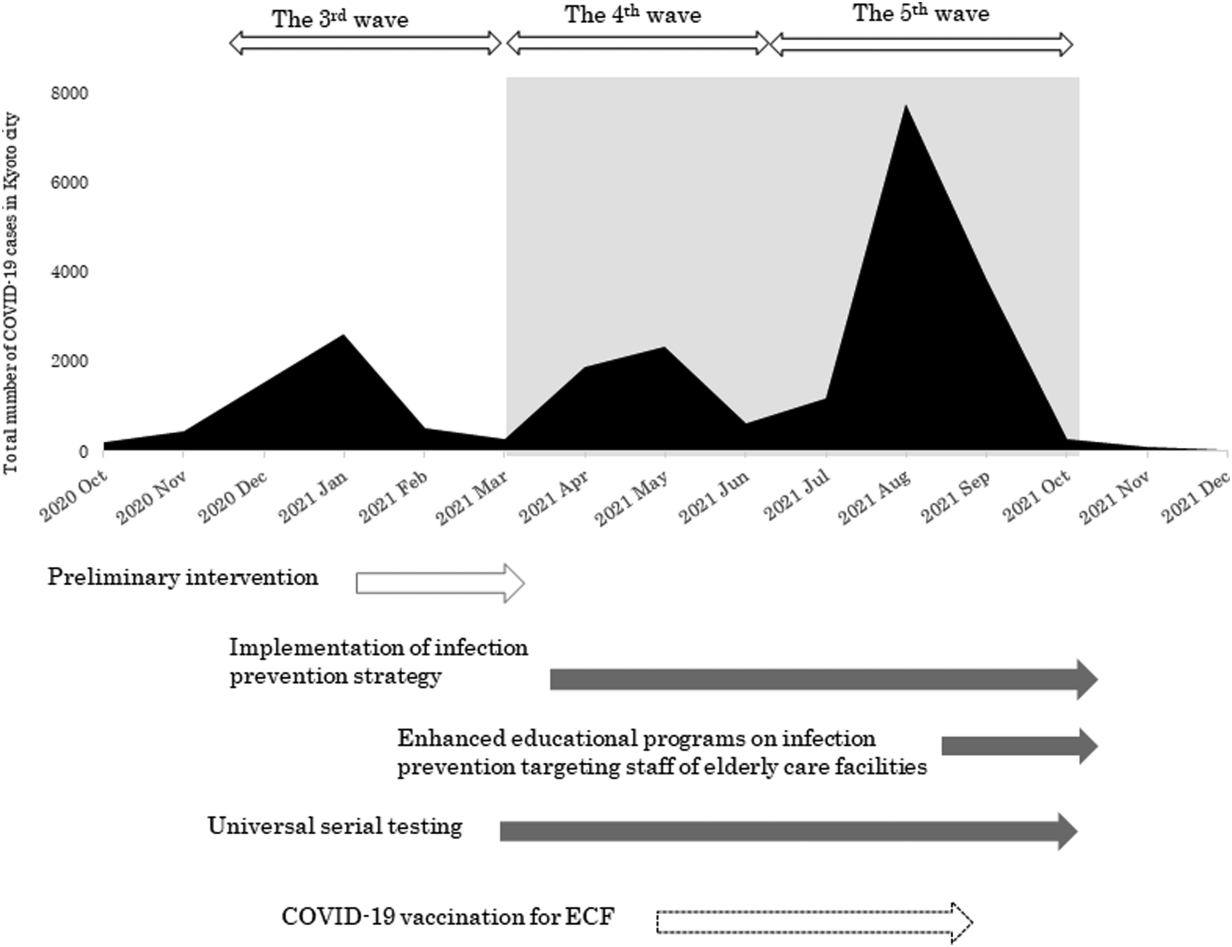
Trend of COVID‐19 cases in Kyoto City and the contents of proactive intervention. Preliminary intervention began in the third wave. Targeted facilities were expanded during the fourth and fifth waves (shaded in grey). Mass testing eventually ended when the fifth wave was over

The trend in the number of COVID‐19 cases during the study period and measures taken are shown in Figure 1. From January 2021, when the third wave peaked, we conducted preliminary interventions for eight ACFs and clarified operational methods and issues. Then, PCR mass testing was launched, targeting the whole facilities. Actual numbers of participating facilities and tests are shown in the Table 1. Of the 1,144 facilities that were subjected to PCR mass testing, over 70% of them participated in the monitoring program. By the end of August 2021, the two‐dose vaccination program involving ACFs was completed. Later, when the fifth wave came to an end, all screening tests were finished. A total of 394,377 mass tests were performed, and 72 (0.018%) were positive, of which 41 were confirmed cases. Ten out of the 41 cases (24.4%) resulted in transmission within facilities.

### 3.2 Trend in the proportion of ECF‐related cases during the study period

**FIGURE 2:**
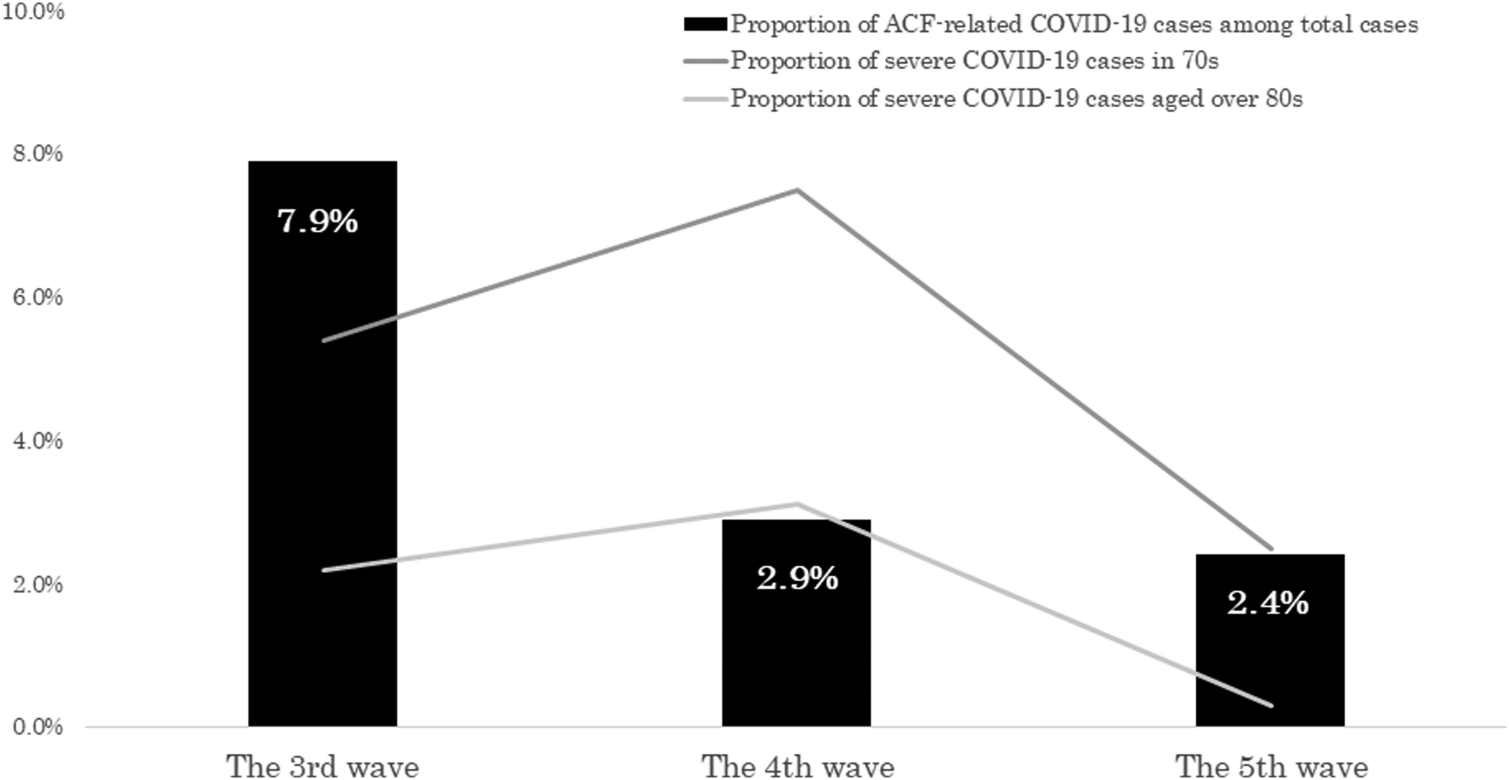
Proportion of ACF‐related COVID‐19 cases and severe cases in the elderly during the study period in Kyoto City, Kyoto, Japan. Proportion of ACF‐related cases and severe cases in the elderly significantly decreased during the study period

The proportion of ACF‐related COVID‐19 cases in total cases decreased from 7.9% in the third wave to 2.9% in the fourth wave and 2.4% in the fifth wave, although the total number of COVID‐19 cases spiked in the fifth wave due to the spread of the delta variant. (Figure 1 and Table 1) The number of clusters in ACFs also decreased from 7.3 episodes/month in the third wave to 2.6 episodes/month and 3.7 episodes/month in the fourth and fifth waves, respectively. The proportion of severe cases in the elderly population also decreased during the study period (Figure 2).

### 3.3 Description of ACF‐related clusters

During the study period, the number of ACF‐related COVID‐19 cases (both residents/users and staff) was 720, including sporadic and cluster‐related cases. Among them, cluster‐related cases comprised 451 cases (staff: 158 cases; seniors: 293 cases) in 42 clusters. Of the 145 staff members whose vaccination history was confirmed, 55 (37.9%) were confirmed to have completed the vaccination program, and of the 155 seniors, 92 (59.4%) had completed the program. There were 19 clusters in which staff were the index cases, and the average duration of clusters was 25.7 days (ranging from 13 to 44 days). Of the 20 clusters that occurred during the period of mass testing, four were discovered by mass testing.

## 4 DISCUSSION

This is the first real‐world data from Japan showing the effect of comprehensive efforts to reduce the spread of COVID‐19 within ACFs. In addition, collaboration with a national university, local authority, and local ACFs is rare in Japan, and this framework will be of immense value in the event of a future pandemic. As a result of this project, clusters in ACFs, the proportion of ACF‐related cases, and severe cases were successfully reduced despite the widespread nature of the delta variant.

There is strong observational evidence supporting the merit of serial universal testing of ACF residents and staff to facilitate rapid identification and containment of potential cases.^2^, ^12^, ^13^ Because there is no test method with 100% sensitivity and specificity, a certain percentage of false‐positives and false‐negatives may occur when mass testing is performed. On the other hand, symptom‐based screening of residents might fail to identify all COVID‐19 cases.^4^, ^14^ In order to overcome the uncertainty of the testing, it is necessary to perform tests using multiple specimens or different testing methods.^15^ However, in the case of mass testing, testing only one specimen is practical due to the complexity of specimen collection and cost. When conducting tests in situations where the pre‐test probability is insufficient, it is always necessary to prepare a safety net similar to that in this study. In addition, considering the increase of cases with previous infection, we need to exercise care when interpreting the results of PCR testing.^16^

In addition to the two initiatives of mass testing and improving the quality of infection control, we also promoted a vaccination program for 40,000 residents and staff at ACFs within 4 months as a shielding measure.^17^ According to previous reports, implementation of vaccination programs contributed to the decline, and they also stated that vaccination should be a central part of a multifaceted strategy that includes other infection‐prevention practices to keep residents in ACFs safe.^11^, ^17^, ^18^, ^19^, ^20^, ^21^ Based on our analysis, about 40% of COVID‐19 cases involving staff and 60% involving users of ACFs were breakthrough infections, but the rate of serious infections was significantly reduced, which may be attributed to the effectiveness of the vaccine.

It has been reported that elderly people lose vaccine antibodies more quickly and that the vaccine is less effective against variant strains.^22^ Furthermore, Hsu et al. previously reported that the number of transmissions from unvaccinated controls was three times higher than from fully vaccinated patients.^22^, ^23^ In our cohort, the number of cases identified within clusters decreased over, time and only one quarter of newly identified cases caused intra‐facility transmission. Considering the weakened immune system of the elderly, booster vaccination began in December 2021 in Kyoto, starting from ACFs and medical institutions. As COVID‐19 cannot be eliminated, it is realistic to overcome forthcoming waves with both booster vaccinations and evidence‐based infection control measures.

Potential limitations of this study were as follows: Because it was not possible to perform screening and confirmatory tests using the same specimen, we could not specify whether some of the screening positives were truly false‐positives. However, in the cases determined to be false‐positives, there was no subsequent transmission within the institutions. In addition, we could not evaluate which policy worked to reduce the infection. As is always true with other infections, multifaceted interventions are needed to contain COVID‐19 transmission.^11^, ^21^ We will continue to collect high‐quality information regarding ACF‐related COVID‐19 cases to verify the optimal strategy to deal with COVID‐19 in such settings. We also believe that it is necessary to verify vaccine coverage at the time of a cluster outbreak and the rate of transmission within a facility, but we were unable to analyze them in this study due to a lack of detailed records.

## 5 CONCLUSION

Numbers of COVID‐19 clusters and severe cases related to ACFs were markedly reduced by implementing a comprehensive response. Further study is needed to determine a strategy that is both optimal and sustainable, which may be tailored to the level of community transmission in the era of vaccination.

## AUTHOR CONTRIBUTIONS

**Miki Nagao:** Conceptualization; formal analysis; funding acquisition; investigation; methodology; project administration; supervision. **Yasufumi Matsumura:** Conceptualization; data curation; formal analysis; funding acquisition; investigation; methodology; project administration. **Masaki Yamamoto:** Conceptualization; funding acquisition; investigation; project administration. **Koh Shinohara:** Investigation; project administration. **Satomi Yukawa:** Investigation; project administration. **Taro Noguchi:** Investigation; project administration. **Yasuhiro Tsuchido:** Investigation; project administration. **Takeshi Ikeda:** Conceptualization; project administration; resources; supervision.

### PEER REVIEW

The peer review history for this article is available at https://publons.com/publon/10.1111/irv.12981.

## Data Availability

The data that support the findings of this study are available from the corresponding author upon reasonable request.
